# An integrated approach to care attracts people living with HIV who use illicit drugs in an urban centre with a concentrated HIV epidemic

**DOI:** 10.1186/s12954-016-0121-2

**Published:** 2016-11-22

**Authors:** S. Fernando, R. McNeil, K. Closson, H. Samji, S. Kirkland, C. Strike, R. Baltzer Turje, W. Zhang, R. S. Hogg, S. Parashar

**Affiliations:** 1 British Columbia Centre for Excellence in HIV/AIDS, St. Paul’s Hospital, 608-1081 Burrard Street, Vancouver, BC V6Z 1Y6 Canada; 2 Faculty of Health Science, Simon Fraser University, Burnaby, Canada; 3 Dalhousie University, Halifax, Canada; 4 University of Toronto, Toronto, Canada; 5 Dr. Peter AIDS Foundation, Vancouver, Canada

**Keywords:** HIV, Injection drug use, Barriers to care, Integrated health care, Support services

## Abstract

**Background:**

People living with HIV (PLHIV) who are also marginalized by social and structural inequities often face barriers to accessing and adhering to HIV treatment and care. The Dr. Peter Centre (DPC) is a non-profit integrated care facility with a supervised injection room that serves PLHIV experiencing multiple barriers to social and health services in Vancouver, Canada. This study examines whether the DPC is successful in drawing in PLHIV with complex health issues, including addiction.

**Methods:**

Using data collected by the Longitudinal Investigations into Supportive and Ancillary health services (LISA) study from July 2007 to January 2010, linked with clinical variables available through the British Columbia Centre for Excellence in HIV/AIDS Drug Treatment Program, we identified DPC and non-DPC clients with a history of injection drug use. Bivariable and multivariable logistic regression analyses compared socio-demographic and clinical characteristics of DPC clients (*n* = 76) and non-DPC clients (*n* = 482) with a history of injection drug use.

**Results:**

Of the 917 LISA participants included within this analysis, 100 (10.9%) reported being a DPC client, of which 76 reported a history of injection drug use. Adjusted results found that compared to non-DPC clients with a history of injection drug use, DPC-clients were more likely to be male (AOR: 4.18, 95% CI = 2.09–8.37); use supportive services daily vs. less than daily (AOR: 3.16, 95% CI = 1.79–5.61); to have been diagnosed with a mental health disorder (AOR: 2.11; 95% CI: 1.12–3.99); to have a history of interpersonal violence (AOR: 2.76; 95% CI: 1.23–6.19); and to have ever experienced ART interruption longer than 1 year (AOR: 2.39; 95% CI: 1.38–4.15).

**Conclusions:**

Our analyses suggest that the DPC operating care model engages PLHIV with complex care needs, highlighting that integrated care facilities are needed to support the multiple intersecting vulnerabilities faced by PLHIV with a history of injection drug use living within urban centres in North America and beyond.

## Background

Globally, there are an estimated 3 million people living with HIV (PLHIV) who inject drugs [1, 2]. Within these populations, issues such as delayed HIV testing, low uptake of antiretroviral therapy (ART), ART treatment interruptions, and the need for management of HIV and HIV-related opportunistic infections are pertinent issues [3]. HIV infection has been shown to increase the risk of overdose-related mortality in people who inject drugs (PWID) [4]. The emergence of supervised injection facilities (SIFs)—locations in which PWID consume pre-obtained illicit drugs under staff supervision—aims to address these health-related challenges by providing sterile syringes to PWID, referrals to primary health services, and emergency care in the event of an overdose [5]. In Vancouver, B.C, the Dr. Peter Centre (DPC) operates under a comprehensive harm reduction approach that includes the availability of nurse-supervised injection services in a room located inside the facility, and the provision of harm reduction supplies (e.g., sterile syringes, alcohol swabs). Serving as a multidisciplinary integrated HIV care facility, the DPC was established to address health disparities faced by underserved PLHIV by providing care and support services (see DPC services in Table 1). All DPC clients must undergo a referral and admission process before accessing DPC services. Eligibility for DPC admission includes HIV positive diagnosis, deteriorating health, and demonstrated need for support in order to maintain independence in the community. While there are demonstrated benefits of a specialized integrated approach to care for PLHIV who inject drugs based on need [6], previous research has not focused specifically on the integration of harm reduction, including SIFs, into these health care environments.

**Table 1:** Dr. Peter Centre Services

Art, music, and recreation, complimentary therapy	
Weekly activities offered including gardening, fitness, yoga, and acupuncture. Therapies allow for opportunities to stimulate self-awareness, self-expression, communication, personal development, and greater self-care	
● Individual art therapy ● Open art therapy ● Group music therapy ● Music jam ● Karaoke ● Performances ● Recreational therapy	● Special events ● Community garden ● Exercise program/room ● Group leisure activities (Bingo, games, activities in the living room) ● Pet visits by volunteers ● Complementary therapies (yoga, Reiki, acupuncture)
Support and counseling	
Counseling services offered to help build emotional strength and support mental health and wellness. Supports for problems surrounding addiction and substance use issues	
● Men’s group ● Women’s evening group ● Indigenous group ● Individual counseling	● Group counseling ● Assistance with housing ● Community service resources ● Scheduling appointments and transportation
Nursing and dietetics	
Nurses provide health assessments, medication assistance, and consultation for symptom management. They also play a key role in helping participants successfully engage in HAART treatment. ART adherence support helps to suppress the HIV virus, as well as prevent new HIV transmissions	
● Medication support ● Wound care health teaching ● Supervised injection services	● Harm reduction supplies ● Foot clinic
Amenities:	
DPC clients have access to a variety of amenities	
● Nap room ● Newspapers ● Showers ● Laundry ● Telephones	● Computers ● Library ● TV room ● Movies
Food and nutrition	
Wholesome nutrient-dense meals and dietary guidance for improved health. Meals are offered twice a day, 7 days a week	
● Breakfast ● Lunch	● Snacks ● Coffee

This study sought to characterize the engagement of PLHIV who use(d) illicit drugs and access the DPC. Specifically, this analysis aimed to understand how an integrated model of care that includes a harm reduction approach could be effective amongst PLHIV marginalized by socio-structural inequities (e.g., homelessness, drug criminalization) both in Vancouver, as well as in other settings.

## Methods

### Recruitment

The Drug Treatment Program at the BC Centre for Excellence in HIV/AIDS is mandated by the provincial government to distribute ART free of charge to all eligible PLHIV. Individuals are entered into the Drug Treatment Program when they are first prescribed ART, and a complete prospective profile of ART is maintained [7]. Individuals enrolled in the Drug Treatment Program who were over the age of 19, residents of BC, and able to provide informed consent were eligible to participate in the Longitudinal Investigations into Supportive and Ancillary health services (LISA) study, the aim of which was to examine the experiences of harder-to-reach PLHIV who have accessed ART in BC. The LISA cohort has been previously described in detail [8].

### Outcome and explanatory variables

Our primary outcome variable was self-reported use of DPC services, ascertained by two questions in the LISA survey: (1) what type of place do you live in right now? (Dr. Peter Centre residence provided as an option), and (2) what three agencies or organizations do you use most regularly? (space provided for participant to name specific agency). Table 2 shows the self-reported socio-demographic and psychosocial variables that were included in this analysis: gender (male vs. female vs. transgender), Indigenous ancestry, housing status (stable [house, apartment] vs. unstable [room in hotel, shelter/hostel, no fixed address, recovery house, the DPC, jail]), relationship status (in a relationship [married, common-law, regular partner, non-regular partner] vs. [single, divorced, separated, widowed]), employment status, use of supportive services (daily vs. <daily), self-reported physician-diagnosed mental health disorder, self perceived health now compared to a year prior to the interview (better vs. worse vs. same), having experienced interpersonal violence (ever vs. never), and history of incarceration (ever vs. never). Table 2 also shows clinical variables retrieved from the Drug Treatment Program included in this analysis: ART interruption lasting longer than 1 year between the first ART date and the time of interview, CD4 cell count at the time of interview, and prescription dispensation period (number of days between the two closest dispense dates before interview date).

**Table 2:** Bivariable and multivariable comparisons of PWID who are clients of DPC vs. non-clients (*n* = 558)

Variable		Non-DPC clients *n* = 482 *n* (%)	DPC Clients *n* = 76 *n* (%)	*P* value	Unadjusted odds ratio (95% confidence interval)	Adjusted odds ratio (95% confidence interval)
Socio-demographic and psychosocial indicators						
Gender	Female Male Transgender	176 (36.51) 300 (62.24) 6 (1.24)	11 (14.47) 64 (84.21) 1 (1.32)	<0.001	1.00 3.24 (1.70–6.16)	1.00 4.18 (2.09–8.37)
Indigenous ancestry	Yes No	163 (33.82) 319 (66.18)	24 (31.58) 52 (68.42)	0.701	–	–
Housing status	Stable Unstable	249 (51.66) 233 (48.34)	38 (50) 38 (50)	0.788	1.00 0.94 (0.58–1.52)	–
Relationship status	In a relationship Single	160 (33.20) 321 (66.60)	13(17.11) 63(82.89)	0.008	–	–
Employment status	Yes No	66 (13.69) 416 (86.31)	4 (5.26) 72 (94.74)	0.039	–	–
Supportive service use	<daily Daily	216 (48.55) 229 (51.46)	17 (23.29) 56 (76.71)	<0.001	1.00 3.09 (1.80–5.31)	1.00 3.16 (1.79–5.61)
Mental health disorder diagnosis	No Yes	153 (31.74) 329 (68.26)	17 (22.37) 59 (77.63)	0.099	1.00 1.61 (0.91–2.86)	1.00 2.11 (1.12–3.99)
Health compared to 1 year ago	Better Worse Same	232 (48.13) 148 (30.71) 102 (21.16)	32 (42.11) 18 (23.68) 26 (34.21)	0.040	–	–
Interpersonal violence	No Yes	96 (19.96) 385 (80.04)	8 (10.67) 67 (89.33)	0.055	1.00 2.09 (0.97–4.49)	1.00 2.76 (1.23–6.19)
Incarceration	Ever Never	351 (72.82) 131 (27.18)	55 (72.37) 21 (27.63)	0.934	1.00 0.98 (0.57–1.68)	–
Clinical indicators						
ART interruption for >1 year	No Yes	220 (45.64) 262 (54.36)	47 (61.84) 29 (38.16)	0.009	1.00 1.93 (1.18–3.17)	1.00 2.39 (1.38–4.15)
CD4 at time of interview	Median (IQR)	300 (190–460)	285 (150–470)	0.602	0.98 (0.88–1.09)	–
Prescription dispense period	Median (IQR)	52 (28–66)	38.5 (26.5–60.5)	0.058	0.93 (0.82–1.07)	–

### Inclusion criteria and statistical analysis

In order to be included in this analysis, participants were required to report a history of ever injecting drugs. A descriptive profile of PWID within the LISA study comparing those who are clients of the DPC vs. those who are not DPC clients was established by conducting the Chi-square test for categorical variables, and the Wilcoxon rank-sum test for continuous variables. Explanatory variables of interest were selected using Akaike information criterion minimization.

## Results

Between July 2007 and January 2010, interviews were conducted with over 1000 participants, of whom 917 had complete clinical data within the Drug Treatment Program. Among 917 LISA participants, 100 (10.9%) were DPC clients. Table 2 compares individuals with a history of injection drug use who are DPC clients (*n* = 76) to individuals with a history of injection drug use who are not DPC clients (*n* = 482).

The results of the bivariable analysis suggest that DPC clients were less likely to be female (14.5 vs. 36.5%; *p* ≤ 0.001), in a relationship (17.1 vs. 33.2%; *p* = 0.008), employed (5.26 vs. 13.7%; *p* = 0.04), and more likely to use supportive services daily (76.7 vs. 51.5%, *p* ≤ 0.001) compared to non-DPC clients. DPC clients were less likely to report their health to be better than a year ago compared to non-DPC clients (42.1 vs. 48.1%; *p* = 0.04). DPC clients were more likely to have ever experienced ART interruption for more than 1 year between the first ART date and interview date (61.8 vs. 45.6%, *p* = 0.009).

Multivariable results found that compared to those who were not DPC clients, DPC clients were more likely to be male compared to female (adjusted odds ratio [AOR]: 4.18; 95% confidence interval [CI]: 2.09–8.37), to use supportive services daily (AOR: 3.16; 95% CI: 1.79–5.61), have been diagnosed with a mental health disorder (AOR: 2.11; 95% CI: 1.12–3.99), have a history of interpersonal violence (AOR: 2.76; 95% CI: 1.23–6.19), and to have ever experienced ART interruption longer than 1 year (AOR: 2.39; 95% CI: 1.38–4.15).

## Discussion

In summary, PLHIV with a history of injection drug use and who report attending the DPC experience more complex health challenges in comparison to those who do not report attending the DPC. A greater number of participants who are clients of the DPC compared to non-DPC clients have experienced interpersonal violence, have ever been diagnosed with a mental health disorder, and have ever experienced HIV treatment interruption. The mutually reinforcing experiences of violence, trauma, and mental health concerns among PLHIV have been documented in the literature as a syndemic [9–11]. This is aligned with our finding that DPC clients with a history of injection drug use are more than twice as likely to have been diagnosed with a mental health condition compared to their non-DPC client counterparts. Untreated mental health disorders can make navigating conventional health care systems arduous, often leaving PLHIV who live with these conditions more likely to experience suboptimal treatment and health outcomes [12]. Syndemic health issues, such as mental health and illicit drug use, interfere with individuals managing their HIV, and engaging in safer practices, thus acting as barriers to adequate care [12]. Therefore, specialized health care services targeting PLHIV with complex health issues are necessary for optimization of health outcomes.

Use of daily support services by DPC clients was approximately three times higher when compared to their non-DPC client counterparts. These findings suggest that integrated healthcare approaches are necessary to support PLHIV with complex health issues, including injection drug use. The DPC provides a range of services appealing to the diverse healthcare needs of clients. Engagement with the facility occurs through multiple entry points (e.g., meal program, medication support) that may ultimately lead to different trajectories of service use to promote overall health, and may improve client retention.

There are several limitations to consider in this study. Results should be interpreted with caution, as the measurement of lifetime history of injection drug use does not necessarily correspond to current drug use. In addition, potential information bias is important to note as socio-demographic and psychosocial indicators were self-reported in the LISA survey.

## Conclusions

In conclusion, DPC clients with a history of injection drug use experience more complex health issues than PWID who are not DPC clients. However, our analyses demonstrate that the DPC integrated model of care helps facilitate access to support services for this population. The DPC’s referral and selection criteria successfully capture and engage key populations experiencing complex health issues. Further research on integrated health care facilities, including harm reduction services, should be conducted to examine whether they improve treatment outcomes and quality of life among key populations living with HIV.

## Abbreviations

DPC: Dr. Peter Centre
PLHIV: People Living with HIV
PWID: People who inject drugs
